# Linkage Mapping and Comparative Genomics of Red Drum (*Sciaenops ocellatus*) Using Next-Generation Sequencing

**DOI:** 10.1534/g3.116.036350

**Published:** 2017-01-24

**Authors:** Christopher M. Hollenbeck, David S. Portnoy, Dana Wetzel, Tracy A. Sherwood, Paul B. Samollow, John R. Gold

**Affiliations:** * Marine Genomics Laboratory, Department of Life Sciences, Texas A&M University, Corpus Christi, Texas 78412; † Mote Marine Laboratory and Aquarium, Sarasota, Florida 34236; ‡ Department of Veterinary Integrative Biosciences, Texas A&M University, College Station, Texas 77845

**Keywords:** RAD-seq, SNPs, genetic map, haplotypes, synteny

## Abstract

Developments in next-generation sequencing allow genotyping of thousands of genetic markers across hundreds of individuals in a cost-effective manner. Because of this, it is now possible to rapidly produce dense genetic linkage maps for nonmodel species. Here, we report a dense genetic linkage map for red drum, a marine fish species of considerable economic importance in the southeastern United States and elsewhere. We used a prior microsatellite-based linkage map as a framework and incorporated 1794 haplotyped contigs derived from high-throughput, reduced representation DNA sequencing to produce a linkage map containing 1794 haplotyped restriction-site associated DNA (RAD) contigs, 437 anonymous microsatellites, and 44 expressed sequence-tag-linked microsatellites (EST-SSRs). A total of 274 candidate genes, identified from transcripts from a preliminary hydrocarbon exposure study, were localized to specific chromosomes, using a shared synteny approach. The linkage map will be a useful resource for red drum commercial and restoration aquaculture, and for better understanding and managing populations of red drum in the wild.

Next generation sequencing (NGS) has provided a set of powerful tools for characterizing the genome of nearly any species. However, genome assembly remains a challenge for organisms with large, repetitive genomes, such as those of many plants and vertebrates (Bradnam *et al.* 2013). For many applications, a suitable alternative to genome sequencing and assembly is construction of a genetic linkage map. While linkage maps traditionally have been possible only for species with pedigree information, or those that could be crossed experimentally, a combination of reduced-representation NGS (Baird *et al.* 2008; Elshire *et al.* 2011) and advances in single-cell genomics (Xu *et al.* 2015) have made it possible to obtain a high-density linkage map for almost any species. High-density linkage maps generated recently have been used to understand the genetic basis of adaptive phenotypic traits (Baxter *et al.* 2011; Henning *et al.* 2014), study the genetic basis of sex determination (Anderson *et al.* 2012; Palaiokostas *et al.* 2013a,b), map quantitative trait loci in economically important species (Chutimanitsakun *et al.* 2011; Houston *et al.* 2012; Talukder *et al.* 2014), and for population and comparative genomics (Amores *et al.* 2011; Hohenlohe *et al.* 2012; Bradbury *et al.* 2013; Manousaki *et al.* 2015).

The red drum (*Sciaenops ocellatus*) is an estuarine-dependent, marine fish distributed throughout the northern Gulf of Mexico (GoM) and along the east coast of the United States (Pattillo *et al.* 1997). The species supports a large recreational fishery in U.S. waters and is reared in captivity for both restoration aquaculture in the U.S. and commercial aquaculture in China, the U.S., and elsewhere (Gold *et al.* 2008; FAO 2015). The species also has been used recently as a model to investigate anthropogenic impacts on marine systems, including studies assessing impacts of agricultural chemicals (Alvarez and Fuiman 2005, 2006) and ocean acidification (Diaz-Gil *et al.* 2015). Following the Deepwater Horizon oil spill in the GoM in 2010, red drum also has been selected as a model to study physiological and genetic effects of acute oil exposure in a marine fish.

Here, we present a dense genetic linkage map for red drum, combining 481 previously mapped anonymous and gene-linked microsatellite loci (Hollenbeck *et al.* 2015) with 1794 haplotyped contigs derived from double-digest restriction-site associated DNA (ddRAD) sequencing. Using the high degree of shared synteny observed between red drum and six other fish species whose genomes are published, we demonstrate how synteny data can be used to infer likely positions of candidate genes, in this case identified from transcripts obtained during a preliminary hydrocarbon exposure study, which could not be mapped using traditional linkage approaches.

## Materials and Methods

Prior to analysis of genetic data, a reduced-representation, reference genome for red drum was constructed. Twenty individuals from across the range of the species, including parents from two mapping crosses, were used to produce a ddRAD library following Peterson *et al.* (2012) as modified by Portnoy *et al.* (2015). The library was sequenced on an Illumina MiSeq DNA sequencer producing 300 bp, paired-end reads. Raw sequencing reads were demultiplexed using the program process_radtags from the *Stacks* package (Catchen *et al.* 2011), and a *de novo* reference genome was assembled using the *dDocent* pipeline (Puritz *et al.* 2014). Because sampled RAD contigs had a mean size of 300 bp, the entire sequence of each RAD contig was recovered during reference assembly. A preliminary annotation of the reference genome with the BLAST algorithm revealed the presence of multi-copy nuclear ribosomal RNA (rRNA) genes. To avoid downstream issues with read mapping caused by multi-copy loci, a custom script was used to remove rRNA contigs, and all contigs with a total length of <150 bp. All further analysis of RAD sequences utilized this reference genome for read mapping and SNP calling.

Tissue samples from two outbred, full-sibling mapping crosses (Family A: *n* = 117; Family B: *n* = 116) used to generate the microsatellite-based linkage map (Hollenbeck *et al.* 2015), were extracted using Mag-Bind Tissue DNA kits (Omega Bio-Tek). RAD libraries were constructed following procedures outlined in Portnoy *et al.* (2015), and sequenced on two lanes of an Illumina HiSeq 2000 DNA sequencer. Raw sequencing reads were demultiplexed, using the program process_radtags, to produce a file containing raw reads for each individual. Read mapping and SNP calling were conducted for each family separately, using the *dDocent* pipeline and the reduced-representation reference genome. Raw SNP genotypes were filtered stringently using the VCFtools package (Danecek *et al.* 2011). First, individual genotypes called from <10 reads were removed, followed by all loci with a mean Phred quality score of <20. An iterative filtering process was then used to maximize the number of individuals and loci in the final dataset. Loci with >50% missing data were excluded, followed by individuals with a mean depth of less than five reads and >95% missing data. Next, loci and individuals with >25% missing data were excluded, and loci with a minor allele frequency <0.05 were removed. The bash script *dDocent_filters* (https://github.com/jpuritz/dDocent/tree/master/scripts) was used to remove loci based on numerous criteria, including mean read depth, ratio of quality to depth, strand representation, allelic balance in heterozygous individuals, and proper read pairing. Complex polymorphisms were then decomposed to individual SNP or indel loci, using the *vcfallelicprimitives* command in the *vcflib* package (https://github.com/vcflib/vcflib). Loci were then collapsed into haplotypes within each RAD contig, using the program *rad_haplotyper* (Willis *et al.* 2016; http://www.github.com/chollenbeck/rad_haplotyper). Briefly, the program uses read alignments to record combinations of SNPs (haplotypes) present across paired-end reads. It also applies filters to flag loci that have an excess of haplotypes (potentially indicative of paralogous loci) or a deficit of haplotypes (potentially indicative of genotyping error), given the SNP genotypes. The program removed loci that were haplotyped successfully in <75% of individuals, and kept indel loci when the indel was the only polymorphism on the contig; other indels were excluded from the analysis to avoid complications in haplotyping. The resulting file for each family contained one diploid genotype per individual for all remaining RAD contigs. After filtering, the dataset for Family A consisted of 72 progeny with genotypes at 786 RAD contigs, and the dataset for Family B consisted of 81 progeny with genotypes at 1340 RAD contigs.

RAD-based genotype data were combined with microsatellite genotype data obtained previously (Hollenbeck *et al.* 2015), and the resulting data file imported into JoinMap (van Ooijen 2012). RAD loci were then added to previously defined linkage groups (Hollenbeck *et al.* 2015). To reduce the chance of incorrectly adding loci to existing linkage groups, an initially conservative LOD score of 9.0 was applied, followed by two more rounds of grouping at an LOD of 6.0 and 3.0. After assigning loci to linkage groups, groups of loci that exhibited an observed recombination rate of zero were identified, and only a single representative locus was retained for initial ordering. Tests for segregation distortion were carried out using chi-square goodness-of-fit tests, implemented in JoinMap. Family-specific maps were generated by applying the multipoint maximum likelihood algorithm for outbred crosses (van Ooijen 2011), as implemented in JoinMap. Marker order for shared loci was compared between families, and incongruities corrected by identifying and removing problematic loci, which most often displayed either segregation distortion or contained probable genotyping errors. Loci excluded initially because of lack of recombination with another locus were then added to family-specific maps by placing them at the same map position as the representative mapped locus. Family-specific maps were combined into a consensus map with the program MergeMap (Wu *et al.* 2011), using equal weights for both families. To investigate whether mapped loci were located in protein coding genes, loci in the consensus map were screened against the NCBI nonredundant nucleotide (nt) database, filtered to include only vertebrate sequences, using the NCBI BLAST+ suite (Camacho *et al.* 2009). Hits were considered a successful match if the *e*-value for the hit was <1 × 10^−10^.

A custom pipeline (https://github.com/chollenbeck/synteny_mapper), written in the Perl programming language, was used to compare the linkage map for red drum to the genomes of six fish species for which chromosome-level scaffolding was available. Genome assemblies were downloaded for stickleback (*Gasterosteus aculatus*; gasAcu1), Nile tilapia (*Oreochromis niloticus*; onil1.1), green spotted puffer (*Tetraodon nigroviridis*; tnig_v8), fugu (*Takifugu rubripes*; FUGU5), European seabass (*Dicentrarchus labrax*; dicLab v1.0c), and barramundi (*Lates calcarifer*; v3). The pipeline first screened all mapped red drum loci for which sequences were available (2252 of 2275) for matches (*e*-value <1 × 10^−10^) to the genome of each comparison species, using the discontiguous megablast algorithm in the BLAST+ suite. Red drum loci with significant matches to multiple chromosomes within a comparison species were discarded to prevent ambiguities related to multi-copy loci. For each locus that matched a single chromosome, the start position of the sequence on the chromosome was recorded. Next, regions of shared synteny, defined as regions consisting of at least two loci that shared a common marker order (collinearity) between the red drum linkage map and the genome of a comparison species, and which are uninterrupted by other mapped loci, were identified. The algorithm used to identify regions of shared synteny takes into account the possibility of small errors in genome assembly and map order, and allows small differences in marker order. In this case, departures from collinearity between loci separated by <2.5% of the total linkage group or chromosome length were tolerated.

By using regions of shared synteny, it is possible to infer positions of unmapped loci, provided that a sequence for the locus is available, and it matches unambiguously to the genome of a comparison species (Hollenbeck *et al.* 2015). To demonstrate the utility of the red drum linkage map as a tool for inferring the genomic position of loci of interest, this approach was applied to an unpublished dataset of candidate genes identified from red drum transcripts obtained in a preliminary hydrocarbon exposure study (dataset doi: 10.7266/N73T9F7J). Using the final module of the *synteny_mapper* pipeline, cDNA sequences of 724 gene transcripts were screened against the genome of each comparison species, using BLAST+, as above. Transcript sequences with single hits to the genome of at least one comparison species were identified, and for each locus it was determined whether the locus was located in a region of shared synteny between the red drum linkage map and the genome of the comparison species. When transcript sequences were located in a region of shared synteny and the region contained more than two mapped loci, the script identified the smallest possible interval within the region into which the locus could be positioned and recorded the nearest left and right flanking loci. The distribution of shared syntenic blocks and inferred positions of synteny-mapped loci, relative to the red drum linkage map, were visualized using *ggplot2* (Wickham 2009) and *circos* (Krzywinski *et al.* 2009).

### Data availability

Unpublished oil exposure data are publicly available through the Gulf of Mexico Research Initiative Information & Data Cooperative (GRIIDC) at https://data.gulfresearchinitiative.org (doi: 10.7266/N73T9F7J). Raw, demultiplexed sequence reads may be found in NCBI’s Short Read Archive (SRA) under accession number PRJNA357008. Additional data files, including final SNP dataset (VCF format) and SNP haplotype dataset (JOINMAP format) for both families can be found in Supplemental Material, File S1. The final reduced representation reference genome can be found in File S2. Scripts to reproduce tables and figures, as well as additional data files, can be found at http://www.github.com/chollenbeck/red_drum_map. 

## Results

Following assembly, the reduced-representation reference genome contained 38,887 RAD contigs, with a mean size of 264.9 bp. Additional filtering for contigs below minimum threshold size (<150 bp), and those containing rRNA sequences resulted in a final reference assembly of 33,865 RAD contigs, with a mean size of 284.6 bp. The total length of the reference sequence was 9,638,003 bp. Assuming a total red drum genome size of ∼810 Mb (Gold *et al.* 1988), the reference covered ∼1.19% of the genome.

After filtering, the dataset for Family A (72 progeny) consisted of 786 RAD contigs (containing 1383 SNPs), and the dataset for Family B (81 progeny) consisted of 1340 RAD contigs (containing 2620 SNPs). The difference in number of usable RAD contigs between families was largely the result of differences in overall DNA quality of individual samples from the two families. A detailed summary of filtering results is presented in Table S1. After combining RAD contig data with microsatellite and EST-SSR genotypes assayed previously from the same individuals, the total mapping dataset consisted of 1218 and 1779 loci for Families A and B, respectively.

**Figure 1:**
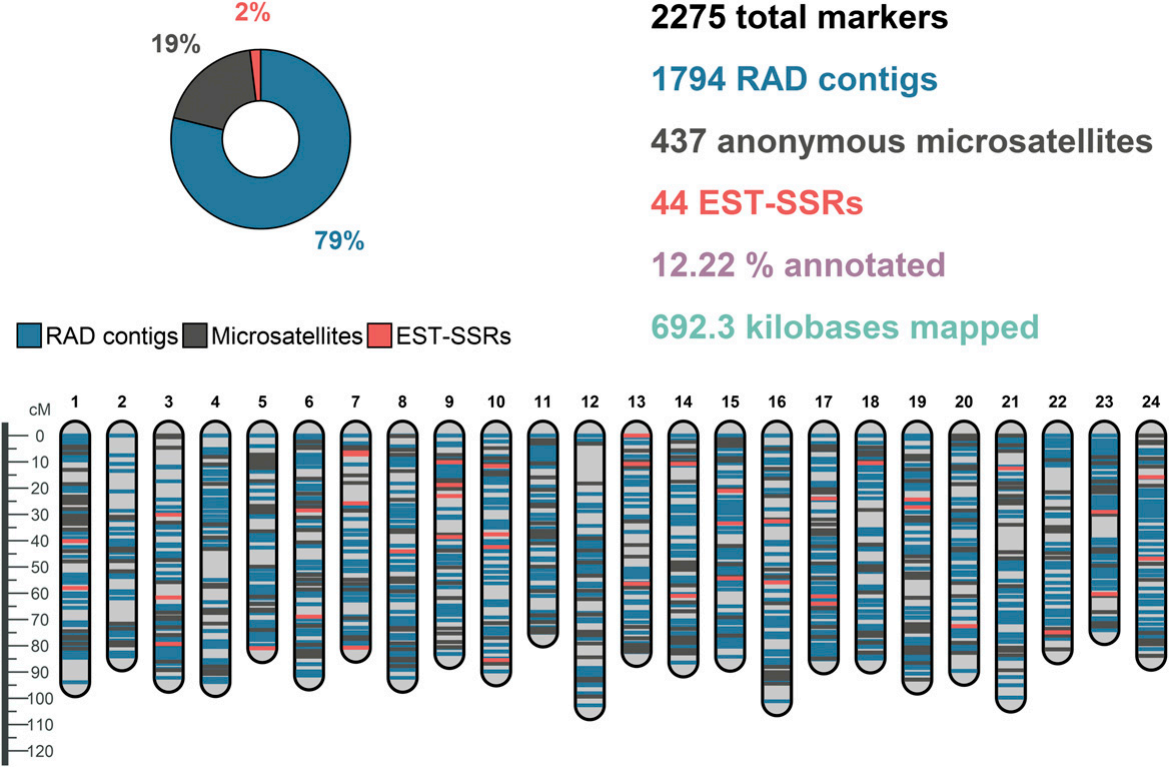
Red drum consensus linkage map.

The consensus linkage map (Figure 1) contained 2275 loci, including 1794 RAD contigs (consisting of 3462 SNP loci), 437 anonymous microsatellite loci, and 44 EST-SSRs; the combined length of mapped fragments totaled 692.3 kb. The mean number of loci per linkage group was 94.79 and the mean marker interval was 0.94 cM. The average length of a linkage group was 87.72 cM; the total map length was 2105.30 cM. Because of the tendency of MergeMap to inflate the total size of linkage groups when combining maps (Khan *et al.* 2012), the average of individual-specific maps may represent a more accurate estimate of total map size. Averaged across individual-specific maps, the mean length of linkage groups and total map length were 71.81 and 1704.40 cM, respectively. Of 2275 total loci, 278 (12.2%) were located directly in coding regions and could be assigned a putative function based on a BLAST search. Summary statistics for the consensus-, family-, and individual-specific maps are presented in Table 1; detailed information regarding map positions and annotations for the consensus linkage map are available in Table S2.

**Table 1:** Summary statistics for red drum linkage maps

	*Consensus*	*Family A*	*Family B*	*AF*	*AM*	*BF*	*BM*
*Total loci*	2275	1001	1569	720	674	961	976
*Microsatellite loci*	437	327	334	238	241	194	190
*EST-SSR loci*	44	33	32	27	25	23	20
*RAD loci*	1794	641	1203	455	408	744	766
*SNP loci*	3462	1170	2456	910	884	1693	1768
*Mean LG size*	87.721	77.735	70.084	75.961	74.102	73.422	63.634
*Mean loci per group*	94.792	41.708	65.375	30	28.083	40.042	40.667
*Mean marker interval*	0.935	1.91	1.087	2.606	2.73	1.869	1.595
*Total map length*	2105.3	1865.7	1682.0	1823.1	1778.4	1762.1	1527.2

Column names represent maps constructed using various subsets of mapping individuals: Consensus, Consensus map with all individuals; Family A, Family-specific map for Family A (male and female); Family B, Family-specific map for Family B (male and female); AF, Family A female; AM, Family A male; BF, Family B female; BM, Family B male. Mean linkage groups (LG) size, mean marker interval, and total map length are measured in centiMorgans.

**Figure 2:**
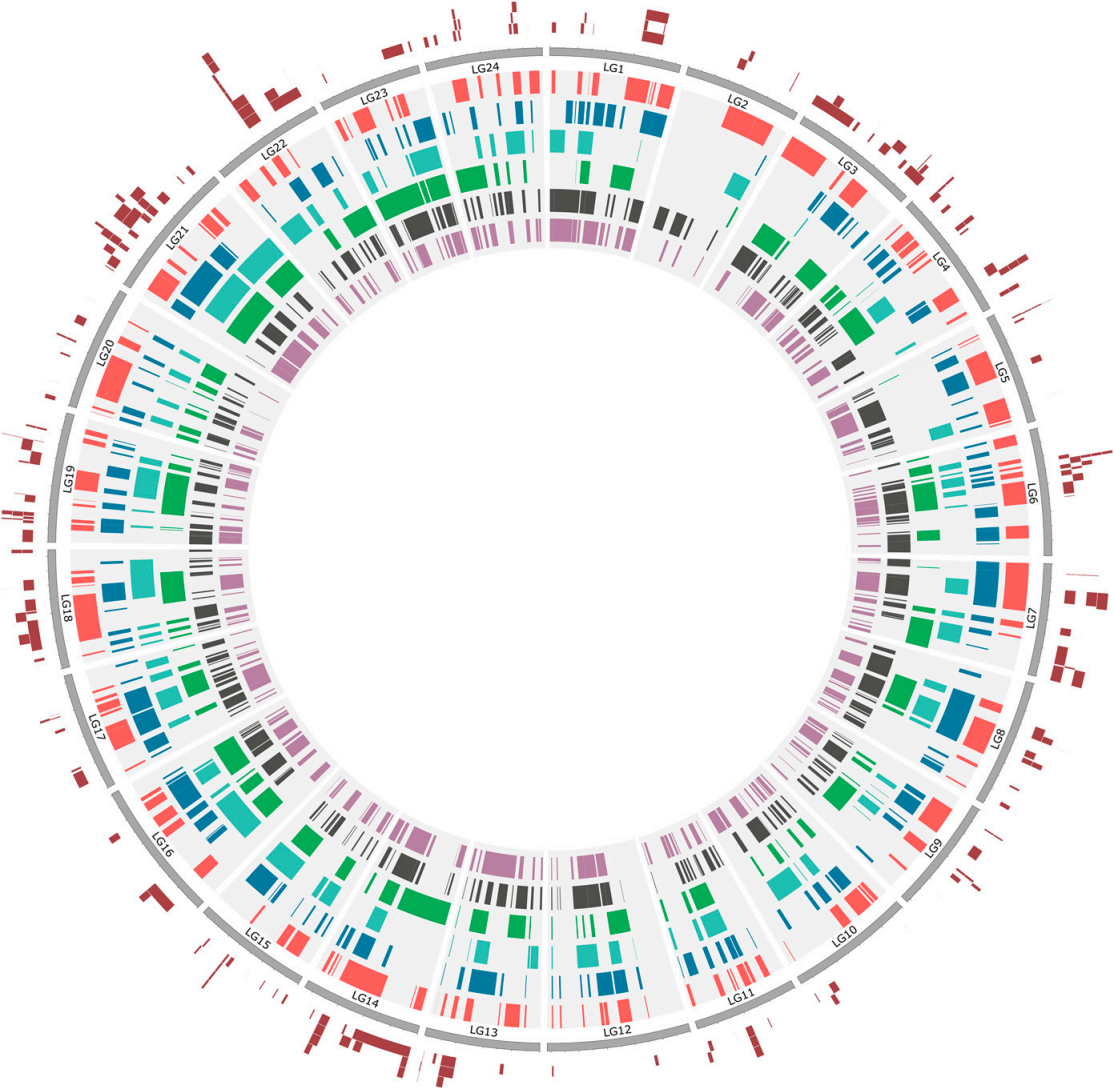
Circular ideogram of shared synteny analysis. Gray rectangles on the outside perimeter represent red drum linkage groups. Colored segments on the inside of the circle represent blocks of shared synteny between the red drum genetic map and genomes of six comparison species: red: stickleback (*Gasterosteus aculatus*); blue: Nile tilapia (*Oreochromis niloticus*); teal: green spotted puffer (*Tetraodon nigroviridis*), green: fugu (*Takifugu rubripes*); dark gray: European seabass (*Dicentrarchus labrax*); purple: barramundi (*Lates calcarifer*). Dark red regions on the outside of the circle represent regions where differentially expressed genes in oil exposure experiments were localized via a synteny-based mapping approach. Putative gene locations that overlap are vertically stacked for clarity.

The total number of loci with significant BLAST hits to single chromosomes ranged from 342 (15.1% of all loci; green spotted puffer) to 1249 (55.3% of all loci; European seabass). The total number of blocks of shared synteny also varied among comparison species, ranging from 87 (green spotted puffer) to 284 (European seabass). The mean number of loci per block of shared synteny was less variable, ranging from 3.047 (stickleback) to 3.196 (fugu). Similarly, while the total size of blocks of shared synteny was highly variable among comparison species, ranging from 92.9 Mb (green spotted puffer) to 259 Mb (Nile tilapia), the proportion of the genome of the comparison species covered by blocks was less variable, ranging from 0.330 (green spotted puffer) to 0.438 (European seabass). Summary statistics for blocks of shared synteny for each of the six comparison species are presented in Table 2; a summary of shared syntenic regions in the form of a circular ideogram displaying blocks for each species relative to red drum linkage groups is presented in Figure 2.

**Table 2:** Summary statistics for shared synteny analysis

*Species*	*Common Name*	*BLAST Hits*	*Number of Blocks*	*Mean Loci per Block*	*Mean Comparison Block Size*	*Mean Map Block Size*	*Total Comparison Block Size*	*Total Map Block Size*	*Proportion of Genome in Blocks*	*Proportion of Map in Blocks*
*Dicentrarchus labrax*	European seabass	1249	284	3.141	0.8	3.247	239.1	922.11	0.413	0.438
*Lates calcarifer*	Barramundi	1156	266	3.192	0.9	3.417	240	908.95	0.409	0.432
*Oreochromis niloticus*	Nile tilapia	736	172	3.163	1.5	4.343	259	747.01	0.394	0.355
*Gasterosteus aculatus*	Stickleback	600	150	3.047	1.1	5.39	167.3	808.44	0.361	0.384
*Takifugu rubripes*	Fugu	399	97	3.196	1.2	9.147	113.6	887.27	0.404	0.421
*Tetraodon nigroviridis*	Green spotted puffer	342	87	3.057	1.1	7.976	92.9	693.92	0.386	0.330

BLAST Hits, number of single BLAST hits to the comparison species genome; Number of Blocks, number of regions of shared synteny containing at least two loci between red drum and each comparison species; Mean Loci per Block, average number of loci in blocks of shared synteny; Mean Comparison/Map Block Size, average size in megabase pairs and centiMorgans of blocks in the comparison species genome and red drum linkage map, respectively; Total Comparison/Map Block Size, total, cumulative size in megabase pairs and centiMorgans of blocks in the comparison species genome and red drum linkage map, respectively; Proportion of Genome/Map in Blocks, proportion of the comparison species genome/linkage map that exists in blocks of shared synteny, calculated based on the total number of base pairs in chromosomal scaffolds and total linkage map size.

**Figure 3:**
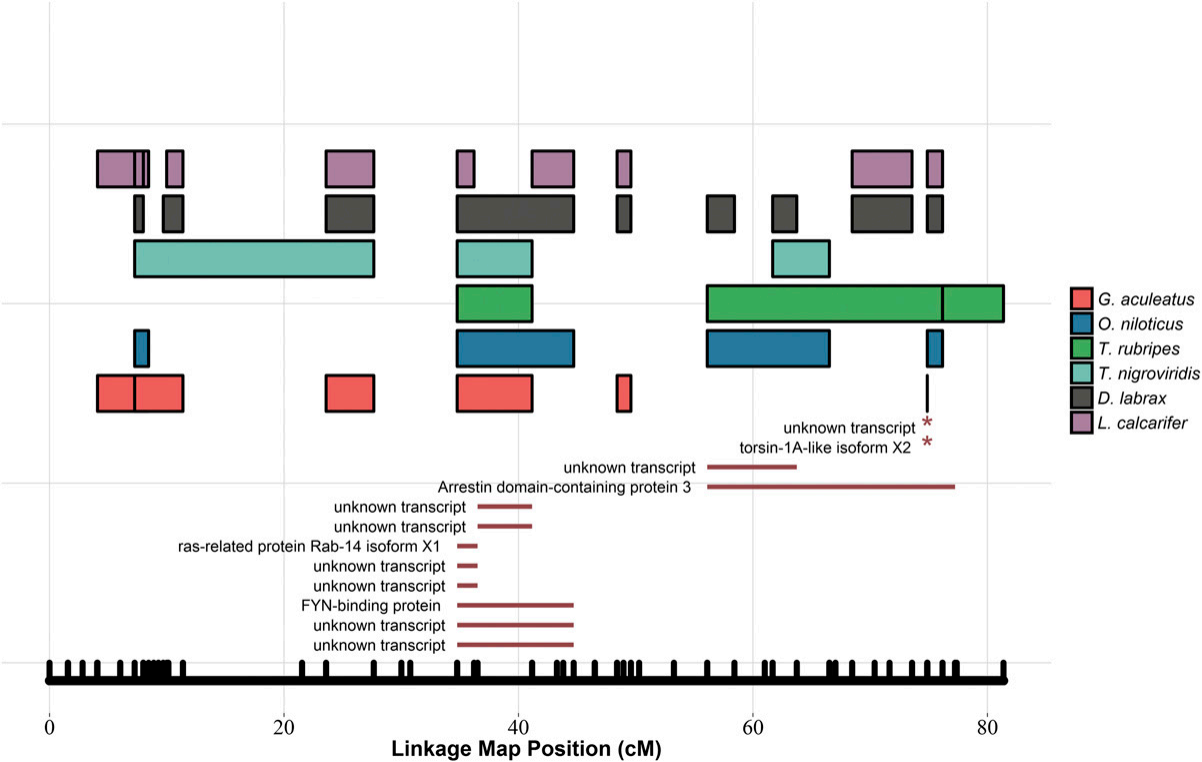
Shared syntenic blocks and synteny-mapped genes on red drum linkage group 22. Colored rectangles represent blocks of shared synteny between the red drum genetic map (horizontal axis) and genomes of six comparison species. Dark-red line segments represent regions where differentially expressed genes in oil exposure experiments were localized via a synteny-based mapping approach. Asterisks denote locations of genes that were synteny-mapped to zero-recombination intervals on the linkage map.

Of 724 candidate gene transcripts identified in oil exposure experiments, 227 (31.4%) were assigned a putative position, using the synteny-mapping strategy (Table S3). Of 277 synteny-mapped loci, 62 (27.3%) were annotated by a BLAST search. Inferred positions of all synteny-mapped genes are presented in Figure 2. The distribution of shared syntenic blocks and synteny-mapped genes on red drum linkage group 22, where eight candidate gene transcripts were localized to a region of ∼10 cM, are shown in Figure 3.

## Discussion

In total, 1794 haplotyped RAD contigs, comprising 3462 SNP loci, were added to the red drum linkage map. Addition of these loci reduced the mean marker interval to <1 cM. The total length of the consensus map (2105.30 cM) is larger than reported previously for the microsatellite-based map (1815.3 cM, Hollenbeck *et al.* 2015); this difference is likely a result of combining maps from different families, using MERGEMAP, which is reported to inflate total map length (Khan *et al.* 2012). Relative to the average total length of individual-based maps reported here, the total length of the consensus map was inflated by ∼24%, consistent with the results of McKinney *et al.* (2015), who found that MERGEMAP increased the size of the Chinook salmon consensus map by ∼30% per additional family. However, there exists a tradeoff between accuracy of map lengths and inclusion of loci on the map (Hollenbeck *et al.* 2015). Because the purpose of our study was to determine the relative positions of loci, rather than the exact frequency of recombination between them, we chose to add more loci to the consensus map by combining information between mapping families.

The RAD-seq linkage mapping process employed here has a number of benefits. First, using long reads from the Illumina MiSeq platform enabled recovery of the entire sequence of each discrete ddRAD contig, thus providing more sequence coverage for comparative genomics and other downstream applications. Second, using haplotypes instead of individual SNPs eliminated redundancy and saved computational time by condensing tightly linked SNPs into single, multi-SNP loci, potentially possessing multiple alleles, thus discriminating more discrete alleles than would otherwise be identified, and, thereby, increasing the number of alleles that segregate in an informative manner (Ball *et al.* 2010). Last, haplotyping allows possible multi-copy loci to be filtered from the dataset because multi-copy loci often display more than two haplotypes within an individual and can be identified on this basis.

Of all comparison species, European seabass, followed by barramundi, had the highest degree of similarity with red drum in terms of total number of homologous loci (established via BLAST search), number and proportion of the genome contained in shared-synteny blocks. The lowest degree of similarity in all three measures was found between red drum and green spotted puffer. Correspondingly, shared-synteny block size was smallest in seabass and barramundi, and largest in fugu and green spotted pufferfish. Finally, in contrast to the considerable variation in the total number of BLAST hits and number of shared syntenic blocks among comparison species, the mean number of loci per block, and the proportion of the comparison species genome in blocks of shared synteny were much more similar among comparisons. There are a number of factors that likely produce this pattern, including the phylogenetic relationship between species, structural changes during genome evolution, and the quality of genome assemblies used in the analysis. European seabass, for example, is more closely related phylogenetically to red drum than green spotted pufferfish (Nelson *et al.* 2016), which likely explains the larger number of BLAST hits in European seabass (1249) as compared to the green spotted pufferfish (342). Having more homologous loci affords the opportunity to detect smaller blocks of shared synteny, as well as the fine-scale rearrangements and translocations that tend to break up larger regions of shared synteny into smaller blocks (Hollenbeck *et al.* 2015).

Results of the synteny analyses were consistent with a high degree of stability in karyotype evolution among teleost fishes (Amores *et al.* 2014). An important implication of this high level of shared synteny is that the information from well characterized genomes can be used to localize candidate genes of interest in nonmodel species. In our study for example, putative locations of previously unmapped candidate gene transcripts were achieved with a success rate of 31.4%, suggesting that the general approach will be useful in localizing candidate genes from environmental impact studies, or other gene expression studies in species for which a complete reference genome is unavailable.

The linkage map described herein will be a valuable resource for studies of red drum and other nonmodel species. Red drum, for example, are cultured both for restoration enhancement and commercial production purposes (Gold *et al.* 2008; FAO 2015). A linkage map can be used to great advantage for commercial aquaculture by enabling the mapping of quantitative traits and facilitating marker-assisted selection for genetic improvement (Liu and Cordes 2004), including the identification of chromosomal regions impacting disease resistance (Houston *et al.* 2012) and sex determination (Palaiokostas *et al.* 2013a,b). In addition, a combination of linkage and linkage disequilibrium (LD) data also has been shown to be effective in detecting changes in contemporary effective population size of wild red drum over time (Hollenbeck *et al.* 2015). High-density linkage maps also can be useful tools for chromosome-level scaffolding in genome assembly (Fierst 2015), and linkage map/genome integrations are increasingly common (Gonen *et al.* 2014; Tine *et al.* 2014). Finally, linkage maps, when combined with population genetics data, can provide a genomic context for loci used in population-level studies to identify genomic islands of adaptation (Bradbury *et al.* 2013) and associations among loci implicated in adaptation (Hohenlohe *et al.* 2012).

## Supplementary Material

Supplemental material is available online at www.g3journal.org/lookup/suppl/doi:10.1534/g3.116.036350/-/DC1.
